# Imaging insights from the bifurcated Y-graft Fontan procedure

**DOI:** 10.1186/1532-429X-18-S1-O115

**Published:** 2016-01-27

**Authors:** Tim Slesnick, W James Parks, Denver Sallee, Sassan Hashemi, Phillip M Trusty, Maria Restrepo, Christopher M Haggerty, Ajit P Yoganathan, Kirk R Kanter

**Affiliations:** 1 grid.189967.80000000419367398Pediatrics, Emory University, Atlanta, GA USA; 2 grid.213917.f0000000120974943Biomedical Engineering, Georgia Institute of Technology, Atlanta, GA USA

## Background

Since 2010, a novel modification to the Fontan procedure has been utilized at our institution where the inferior vena cava / hepatic veins are connected to the branch pulmonary arteries (PA) using a commercially available bifurcated Y-graft. This anatomy presents unique challenges for non-invasive imaging. We sought to evaluate our experience imaging these patients.

## Methods

All patients' medical records were retrospectively reviewed. Echocardiography, cardiac magnetic resonance (CMR) and computed tomography angiography (CTA) images were analyzed by a single reviewer. Post-operative anatomy and dynamic physiologic assessments were performed.

## Results

**Figure 1:**
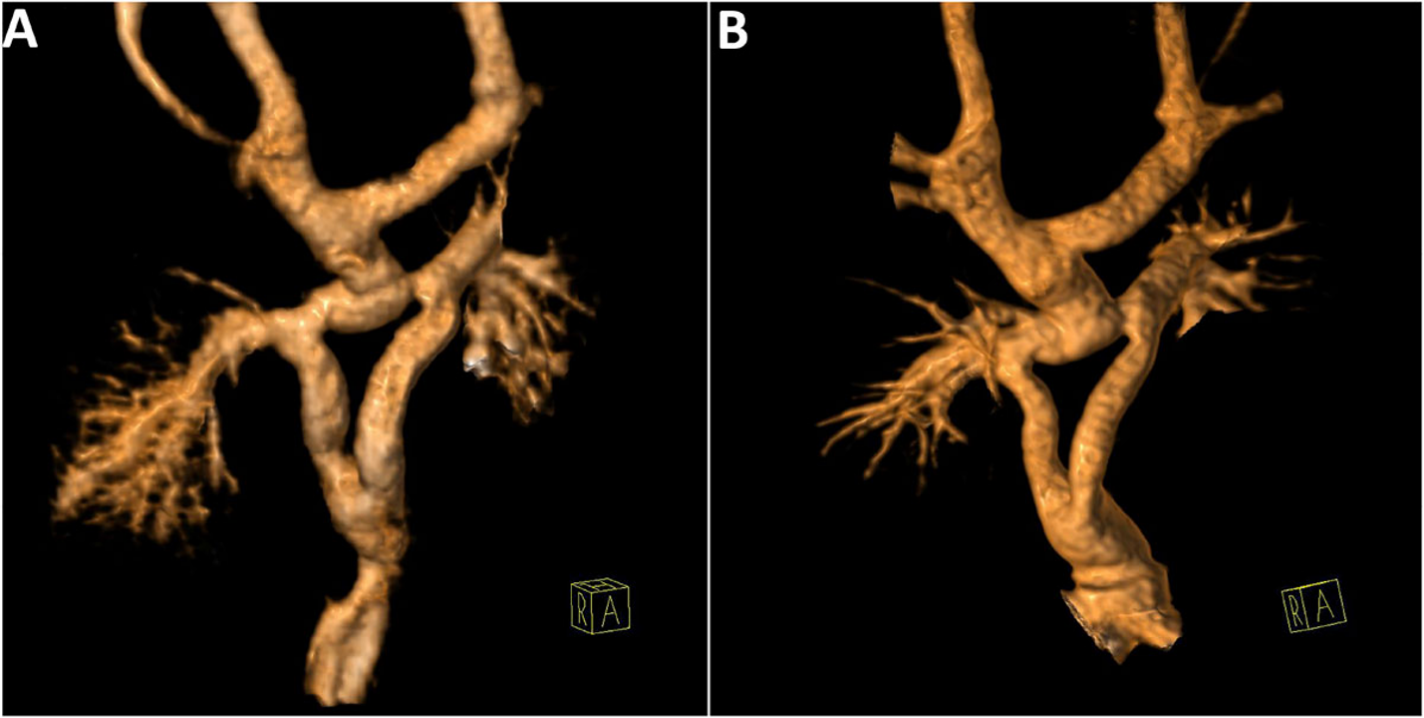
**Characteristic images of Fontan Y-baffles by time-resolved contrast enhanced magnetic resonance angiography (a) and by post-contrast 3D respiratory navigated inversion recovery gradient echo imaging on the same patient on a subsequent scan (b)**. Though both demonstrate the Y-arm baffles and the branch pulmonary arteries well, the inferior Fontan is less well seen on the contrast enhanced magnetic resonance angiography due to swirling flow.

**Figure 2:**
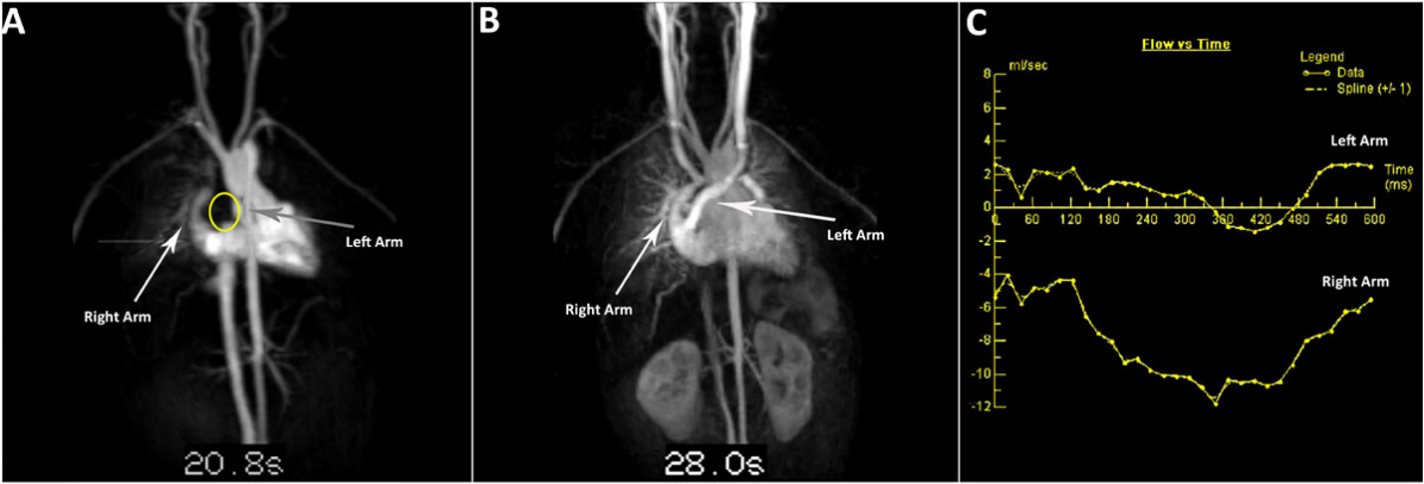
**A patient with bilateral bidirectional Glenn anastomoses and central branch pulmonary artery stenosis with abnormal streaming of their systemic venous return**. With a lower extremity injection, on first pass time-resolved contrast enhanced magnetic resonance angiography, only the right Y-arm is opacified and the left Y-arm is not seen (a), while on a later phase when the left Glenn fills, the left Y-arm is seen to fill retrograde (b). Phase contrast imaging across the Y-arms (c) demonstrates that flow is in opposite directions (antegrade in the right arm and retrograde in the left arm).

From August, 2010 to July, 2015, 45 children (median age 3.6 years, range 1.5 - 18.9 years) have undergone a Y-graft Fontan. In all 45 patients, echocardiography was unable to visualize the Y-arm connections to the branch PA's. Thirty-nine patients underwent CMR a median of 9 days after Fontan (range 4-295 days), and 6 patients with pacemakers underwent CTA. Early in the experience, time resolved contrast enhanced magnetic resonance angiogram (CEMRA) provided the best spatial resolution for baffle evaluation (Figure 1a). In late 2013, our CMR protocol changed to include a blood pool Gadolinium contrast agent, Gadofosveset Trisodium. Subsequently, post-contrast, 3D, respiratory navigated, inversion recovery gradient echo imaging (3D IR GRE) provided superior spatial resolution for the final 19 children (Figure 1b). In one patient, thrombosis in the baffle was found. In 40 patients, some degree of stenosis was found in a Fontan baffle or branch PA, though most were mild (n = 27). Among those with moderate or severe stenosis, most occurred in the central PA, between the insertion of the bidirectional Glenn (BDG) and a Y-arm (n = 7). Phase contrast imaging and the time resolved CEMRA provided insights into Fontan hemodynamics. In 14 patients, inferior systemic venous return was affected by the BDG position and angulation, with competitive flow in either the left or right Y-arm resulting in asymmetric inferior systemic venous return to the branch PA's (Figure 2). This finding was more pronounced in patients with central PA stenosis.

## Conclusions

Cross sectional imaging is essential in children undergoing bifurcated Y-graft Fontan, as the Y-arm insertions onto the branch PA's cannot be reliably visualized by echocardiography. A combination of phase contrast, time resolved CEMRA, and 3D IR GRE CMR imaging provide optimal anatomic and hemodynamic evaluation. Mild graft and PA stenoses were common, but typically had minimal affect on distribution of inferior systemic venous flow. Patients with central branch PA stenosis are more susceptible to adverse streaming of the BDG into a branch Y-arm resulting in competitive flow.

